# Design of a Transformer Oil Viscosity, Density, and Dielectric Constant Simultaneous Measurement System Based on a Quartz Tuning Fork

**DOI:** 10.3390/s24092722

**Published:** 2024-04-24

**Authors:** Hao Yang, Shijie Chen, Jiafeng Ding

**Affiliations:** School of Electronic Information, Central South University, Changsha 410083, China; 15211166381@163.com (H.Y.); csj_chenshijie@163.com (S.C.)

**Keywords:** simultaneous measurement system, quartz tuning fork, transformer oil, impedance analysis

## Abstract

Transformer oil, crucial for transformer and power system safety, demands effective monitoring. Aiming to address the problems of expensive and bulky equipment, poor real-time performance, and single parameter detection of traditional measurement methods, this study proposes a quartz tuning fork-based simultaneous measurement system for online monitoring of the density, viscosity, and dielectric constant of transformer oil. Based on the Butterworth–Van Dyke quartz tuning fork equivalent circuit model, a working mechanism of transformer oil density, viscosity, and dielectric constant was analyzed, and a measurement model for oil samples was obtained. A miniaturized simultaneous measurement system was designed based on a dedicated chip for vector current-voltage impedance analysis for data acquisition and a Savitzky–Golay filter for data filtering. A transformer oil test platform was built to verify the simultaneous measurement system. The results showed that the system has good repeatability, and the measurement errors of density, viscosity, and dielectric constant are lower than 2.00%, 5.50%, and 3.20%, respectively. The online and offline results showed that the system meets the requirements of the condition maintenance system for online monitoring accuracy and real-time detection.

## 1. Introduction

Transformer oil is a commonly used insulating medium in ultra-high voltage power transformers, which, in addition to insulation, also has important functions such as cooling, heat dissipation, and arc suppression [1,2]. Usually, viscosity (ρ), density (η), and dielectric constant (ε) are important characteristic parameters that describe the performance of transformer oil. Specifically, viscosity reflects its insulating properties and liquid flow resistance, and the appropriate viscosity can ensure that the oil plays an ideal cooling effect in long-term operation [3]; density reflects its degree of aging and the safety of the electrode site [4]; and dielectric constant reflects its degree of deterioration and insulating properties [5]. By monitoring these characteristic parameters of transformer oil, nondestructive testing and condition maintenance of transformers can be realized to guarantee the safe operation of power systems [6,7,8,9].

Traditional methods of measuring transformer oil viscosity and density include fluid-based calculations, ultrasound, and Raman spectroscopy, but these methods have some limitations, such as requiring the use of expensive and bulky equipment, only being able to monitor one or two parameters at the same time, and not being able to fulfill the need for real-time monitoring [10]. In recent years, resonant sensors have been introduced for the measurement of fluid density and viscosity. For example, Ghanbari et al. used MEMS sensors to measure the density and viscosity of fluids [11], Heinisch used electromagnetically driven torsional resonators for viscosity and mass density measurements [12], and Ruiz-Díez et al. investigated cantilevered resonators in the shape of tiles for monitoring liquid media [13].

Simple and common tuning fork resonators have been used for a variety of aspects of measurements, e.g., Patimisco et al. reported the design and realization of a spectrometer using a quartz tuning fork [14], Liu et al. used a new quartz tuning fork with a low resonance frequency for a high-sensitivity light-induced thermoelectronic spectroscopy (LITES) sensor [15], in addition to the quartz tuning forks that can be used for density or viscosity measurements [16,17]. Liu et al. chose a steel tuning fork as the sensitive element and achieved the measurement of liquid viscosity and density by deriving the relationship between the resonance frequency and quality factor and the liquid viscosity and density [18]. Voglhuber-Brunnmaier et al. applied quartz tuning fork sensors for the measurement of fuel mixing ratios, engine oil condition monitoring, and particle size characterization of suspensions and analyzed the performance of the measurement system [19]. Sun et al. used quartz tuning fork sensors to measure the viscosity and density of engine oil, and then predicted the remaining oil life and optimized oil change intervals [20]. In addition to conventional capacitive sensors, quartz crystal resonators have been introduced for the measurement of engine oil dielectric constant [21], but not for the measurement of transformer oil dielectric constant.

Quartz tuning forks have the advantages of low cost, high thermal and mechanical stability, fast response speed, simple circuit design, etc. They can be utilized not only in traditional temperature and force sensors, but also in the online monitoring of liquid properties. To date, research has utilized quartz tuning forks in the simultaneous measurement of viscosity and density. However, the measurement of dielectric constant is also critical to the safe operation of transformers, so the development of a device that can simultaneously measure the three parameters of a transformer is vital. The feasibility of a quartz tuning fork three-parameter measurement was investigated by this project team in an earlier study [22]. Building on this foundation, this paper designs a measurement system for simultaneous measurement of three parameters of transformer oil density, viscosity, and dielectric constant, which provides a real-time, low error, and miniaturized solution for monitoring transformer oil characteristics.

## 2. Modeling of Quartz Tuning Fork Measurement System in Transformer Oil

**Figure 1:**
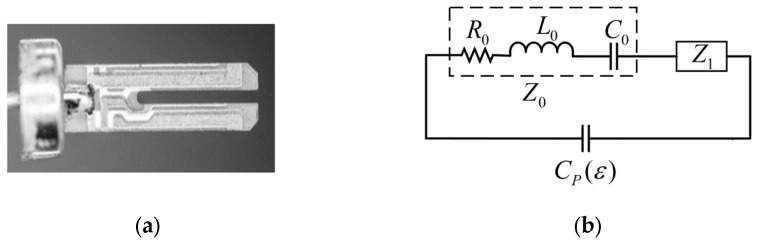
(**a**) Quartz tuning fork with the shell removed; (**b**) equivalent circuit of quartz tuning fork in transformer oil.

After removing the shell, the internal structure of a 32.768 kHz crystal oscillator widely used in the market is shown in Figure 1a, which consists of a combination of two parts: the tuning fork arm and the base. When the quartz tuning fork is placed in a liquid and excited to start oscillating, its bent resonant motion is affected by the damping effect of the liquid environment. This damping comes from two additional stress components applied by the liquid to the tuning fork arm: a normal component, perpendicular to the direction of vibration of the tuning fork arm, and a tangential component, parallel to the direction of vibration of the tuning fork arm. The presence of these two stress components causes the resonant frequency of the quartz tuning fork in liquid to change and no longer equal its intrinsic frequency in air. This change in frequency can be measured and analyzed to reveal certain important physical properties of the liquid.

Using transformer oil as an example, we can equate it to a complex circuit model when a quartz tuning fork oscillates in it. This equivalent circuit is shown in Figure 1b [23,24], which consists of two parts connected in parallel: one part is the static capacitance between the electrodes, which represents the electrostatic interaction between the tuning fork and the liquid, and the other part is a dynamic series branch, which consists of three elements, the equivalent resistance $R_{0}$, the equivalent inductance $L_{0}$, and the equivalent capacitance $C_{0}$, as well as additional impedance caused by the liquid in series. These three elements characterize the friction loss, the added mass, and the elastic effect of the tuning fork as it oscillates in the liquid, respectively. By analyzing and measuring this equivalent circuit in detail, important parameters such as density, viscosity, and dielectric constant of the transformer oil can be accurately obtained.

The expression for the additional impedance $Z_{1}\left(\omega \right)$ introduced by the surrounding liquid is shown in Equation (1):

(1) $$Z_{1}\left(\omega \right)=i\omega M\rho +N\sqrt{\omega \rho \eta }\left(1+i\right)$$

where ρ and η denote the density and viscosity of the liquid, respectively, and ω is the excitation angular frequency. The coefficients M and N, which are closely related to the geometry and oscillation mode of the quartz tuning fork, determine the specific values of normal and tangential stresses [24]. In addition, the static capacitance is affected by the characteristics of the electric field of the surrounding liquid [25]. The electrostatic capacitance expression for a liquid environment is shown in Equation (2):

(2) $$C_{P}\left(\varepsilon \right)=C_{P}+\left(\varepsilon -1\right)\frac{dC_{P}}{d\varepsilon }$$

where $C_{P}\;(\varepsilon )$ is the static capacitance in the liquid environment, $C_{P}$ is the static capacitance in the air, ε denotes the dielectric constant of the liquid, and $dC_{P}/d\varepsilon$ represents the extent to which changes in the electrical characteristics of the liquid environment affect the static capacitance.

When the quartz tuning fork is placed in the transformer oil, its admittance equation can be expressed by Equation (3):

(3) $$Y=\frac{1}{R_{0}+N\sqrt{\rho \eta \omega }+i\left(\omega L_{0}-\frac{1}{\omega C_{0}}+\omega M\rho +N\sqrt{\rho \eta \omega }\right)}+j\omega \left(C_{P}+\left(\varepsilon -1\right)\frac{dC_{P}}{d\varepsilon }\right)$$

This formula incorporates the liquid density, viscosity, and dielectric constant, showing the connection between the admittance spectroscopy and these liquid properties.

In order to accurately determine the characteristic parameters of the fluid, it is necessary to use a standard oil sample for calibration to obtain specific values for the parameters M, N, and $dC_{P}/d\varepsilon$. A transformer oil sample 1 with known characteristic parameters is selected. Its conductivity spectrum is measured, and calibration at the series resonance point is performed to obtain M, N, and $dC_{P}/d\varepsilon$ constants as shown in Equations (4)–(6) [22].

(4) $$M=\frac{1/\omega_{k}C_{0}-\omega_{k}L_{0}-\left(R_{k}-R_{0}\right)}{\omega_{k}\rho_{1}}$$

(5) $$N=\frac{R_{k}-R_{0}}{\sqrt{\rho_{1}\eta_{1}\omega_{k}}}$$

(6) $$\frac{dC_{P}}{d\varepsilon }=\frac{B_{k}/\omega_{k}-C_{P}}{\varepsilon_{1}-1}$$

where $\rho_{1}$, $\eta_{1}$, and $\varepsilon_{1}$ are the viscosity, density, and dielectric constant of the transformer oil sample 1, respectively, and $\omega_{k}$, $R_{k}$, and $B_{k}$ are the angular frequency, equivalent resistance, and susceptance at series resonance.

After determining the values of $C_{0}$, $C_{P}$, $R_{0}$, $L_{0}$, M, N, $dC_{P}/d\varepsilon$, the quartz tuning fork can be put into other transformer oil samples to be tested, measuring its conductivity spectrum and extracting the parameters of the series resonance, and then finally derive the expressions for the three parameters of the transformer oil in the oil samples to be tested:

(7) $$\rho =\frac{1/\omega_{s}C_{0}-\omega_{s}L_{0}-\left(R_{s}-R_{0}\right)}{M\omega_{s}}$$

(8) $$\eta =\frac{M{\left(\left(R_{s}-R_{0}\right)/N\right)}^{2}}{1/\left(\omega_{s}C_{0}\right)-\omega_{s}L_{0}-\left(R_{s}-R_{0}\right)}$$

(9) $$\varepsilon =\frac{C_{P}\left(\varepsilon \right)-C_{P}}{dC_{P}/d\varepsilon }+1$$

where $\omega_{s}$ is the series resonant angular frequency of the quartz tuning fork in the transformer oil to be measured, and $R_{s}$ is the equivalent resistance at resonance in the transformer oil to be measured. These three parameters can be extracted from the conductivity and susceptance spectra.

Based on the above model, the simultaneous measurement of ρ, η, and ε can be performed as long as the frequency-impedance curve of the tuning fork in the fluid to be measured and the frequency-phase curve can be obtained.

## 3. Measurement System Design

### 3.1. Hardware

Impedance tests include the vector current-voltage method, network analysis method, resonance method, and automatic balanced bridge method. The impedance measurement range, applicable frequency range, and advantages and disadvantages of these four methods have been analyzed and compared in Table 1 [26].

**Figure 2:**
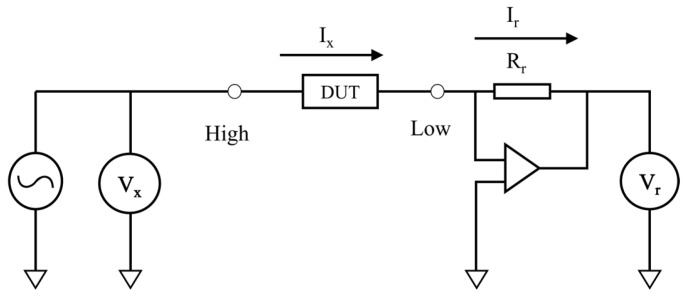
Vector current-voltage schematic.

The vector current-voltage method calculates the unknown impedance by measuring the current and voltage flowing through the part to be measured, and can simultaneously measure impedance and phase. The method has a suitable frequency range and can measure impedance up to nearly 100 MHz, as well as giving high accuracy, and the implementation process is relatively simple, which can facilitate the design of miniaturized embedded measurement systems that can meet the requirements of quartz tuning fork measurement. A schematic diagram for this method is shown in Figure 2. 

An AC voltage source provides sinusoidal signal excitation. At the front end of the component under test, a voltmeter is installed to measure the input voltage $V_{x}$, and a proportional amplifier circuit is set up after the generated current $I_{x}$ flows through the part to be measured. The feedback resistor is $R_{r}$, the proportional amplifier outputs a voltage signal, and a second voltmeter $V_{r}$ is placed at the end to measure the output voltage. The current does not pass through the amplifier and the current $I_{x}$ flows directly through the feedback resistor, so the current $I_{r}$ in the feedback resistor is equal to $I_{x}$. The value of the feedback resistor is known, and the impedance value of the component to be measured is obtained by measuring two voltage values. Using a vector voltmeter, the phase offset between the two voltages can be measured. The offset phase is the phase of the component to be measured.

**Figure 3:**
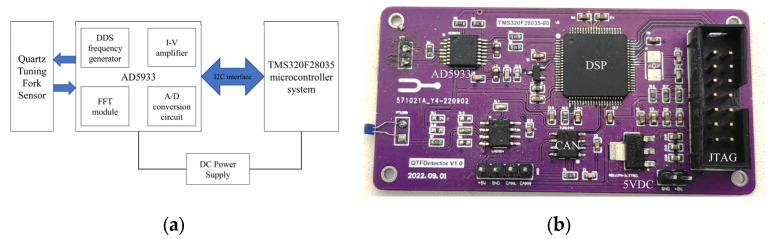
(**a**) Design of hardware circuit of the measurement system; (**b**) hardware boards.

The hardware structure design of the system is shown in Figure 3a. The system includes a TMS320F28035 microcontroller system control circuit and an impedance measurement circuit based on the AD5933 impedance measurement chip.

Considering the huge amount of data involved in real-time monitoring and data analysis processing and the relatively complex signal processing logic, the digital signal processor TMS320F28035 is chosen as the core microcontroller. This controller not only has excellent power consumption performance, but also supports a variety of communication protocols, such as UART (Universal Asynchronous Receiver/Transmitter), I2C (Inter-Integrated Circuit), and CAN (Controller Area Network), making the communication with external devices more flexible and diverse. Its high clock frequency of up to 60 MHz ensures fast data processing capability and response speed to meet real-time requirements.

The AD5933 chip adopts the impedance measurement principle of the vector current-voltage method, and interacts with the microcontroller through the I2C interface for data transmission and control commands. It integrates a DDS (Direct Digital Frequency Synthesis) frequency generator, IV amplifier, A/D converter circuit, FFT module, and low-pass filter circuit, which can realize impedance measurement based on the vector current-voltage method in a highly integrated environment, and can satisfy the requirements of the measurement system in terms of frequency, impedance measurement range, and temperature. Compared to other impedance measurement chips, such as the AD5934, the AD5933 outperforms the AD5934 in terms of data sampling speed, an important advantage in real-time or high-speed impedance measurement applications.

The hardware circuit board of the designed system is shown in Figure 3b and the size is designed to be 70 mm × 35 mm, which makes it easy to carry and suitable for use in various environments. Such size optimization not only enhances portability, but also ensures that it can be easily integrated into various existing devices or systems in practical applications.

The board’s voltage input is 5VDC, which powers the AD5933 chip with a power consumption of less than 0.2 W. The microcontroller is powered by the AMS1117’s step-down 3.3 V, which also keeps its power consumption low, at no more than 0.3 W.

The board is configured with two interfaces, JTAG (Joint Test Action Group) and CAN, to meet different communication needs. The JTAG interface is used to download the program and real-time debugging, and can also read the key data during program running. The CAN interface is used to send the final data, which has the advantages of high speed, long distance, and high reliability.

### 3.2. Firmware

**Figure 4:**
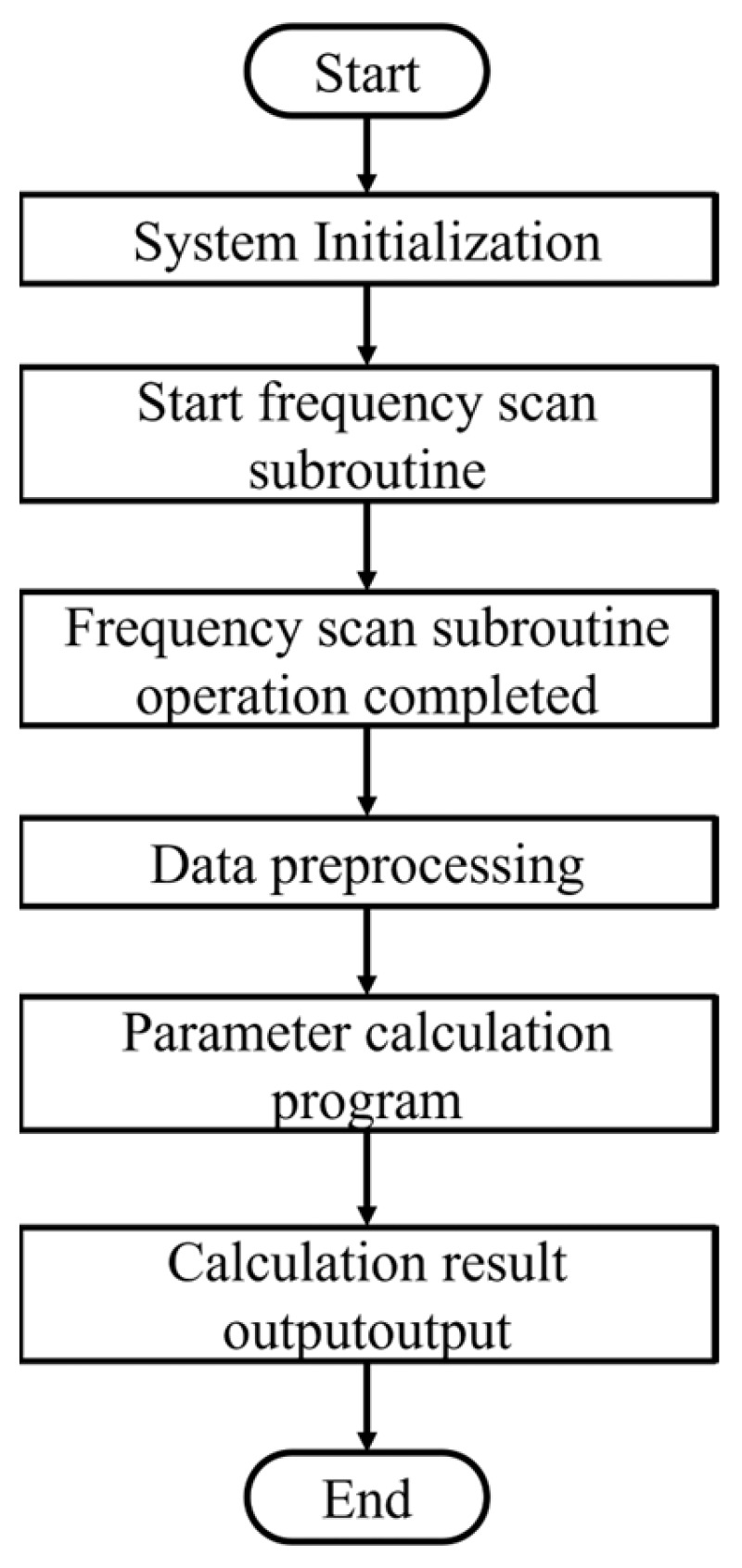
The main program of the control system.

The microcontroller is responsible for controlling the impedance unit in order to collect the impedance and conductance data from the quartz tuning fork. Once the data collection is complete, the microcontroller immediately enters the data processing mode and performs a series of complex arithmetic operations and processing on this raw data. The processed data is then uploaded to the host computer for further analysis and application. The whole software flow is shown in Figure 4. The control system program includes several modules such as system initialization, control subroutine frequency scanning, parameter calculation, and data output.

#### 3.2.1. System Initialization

During the initialization phase, the system performs self-tests and loads configuration parameters to ensure that all hardware is in optimal working condition. Then, the frequency scanning subroutine starts to execute.

#### 3.2.2. Frequency Scan Subroutine

Each time the frequency sweep subroutine is started, four repetitions of the sweep are performed at the specified frequency point. After completing the four scans, median filtering is applied to the data. The advantage of a median filtering technique is that it is particularly suitable for eliminating noise and random fluctuations in the data. By selecting intermediate values, the system is able to automatically exclude anomalous data that might result from external disturbances or internal noise, such as maximum and minimum values. Subsequently, averaging these two intermediate values can further smooth the data and reduce the impact of random errors on the final result. Ultimately, one impedance data output and one phase data output are produced for each frequency point, providing the basis for subsequent parameter calculations.

#### 3.2.3. Data Preprocessing

**Figure 5:**
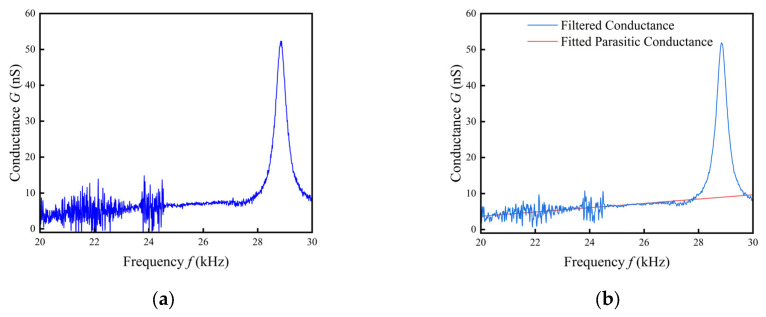
(**a**) Original conductance spectrum; (**b**) conductance after filtering and parasitic conductance.

At the end of the scan, the preprocessing program commences. Figure 5a shows the collected raw conductivity spectrum. Usually, the interference between the sensor leads and the inhomogeneous viscous force of the liquid viscosity on the sensors make the raw data have more burrs, which brings interference in the subsequent calculations, so filters are chosen to improve the data stability.

Because of the need to obtain the maximum conductance, the conductance curve is filtered by the Savitzky–Golay (SG) filter [27], which has the advantage of preserving the relative extremes of the data to the greatest extent possible and allowing the parameters to be freely adjusted to suit different situations. The focus of the Savitzky–Golay filter is on choosing the appropriate window width and the fitting order. In this paper, an SG filter with a window width of 13 and a fitting order of 2 was selected to filter the conductivity curve, which successfully removes the burrs while retaining the extreme values.

The weak electrical conductivity of the liquid itself produces a parasitic conductance, and the more conductive it is, the greater its parasitic conductance. In order to obtain the true change in resistance of the tuning fork monitor due to the viscosity of the liquid, the parasitic conductance needs to be subtracted. 

After filtering the data, the non-resonant portion of the data segment is selected for least squares curve fitting. Since the parasitic resistance is much smaller than the tuning fork resistance at non-resonance, the parasitic conductance is much larger than the tuning fork conductance, and the non-resonant part can be regarded as consisting entirely of parasitic conductance. In the experiment, the 20–23 kHz non-resonant data was selected for curve fitting, as shown in Figure 5b. In the figure, the blue curve is the filtered conductance and the red line is the fitted parasitic conductance.

#### 3.2.4. Parameter Calculation

**Figure 6:**
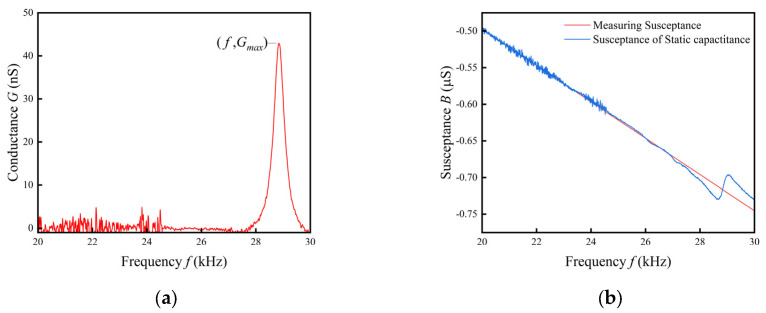
(**a**) Measurement conductivity and parasitic conductivity; (**b**) susceptance curves and fitted straight lines.

Figure 6a shows the conductance curve after preprocessing. By taking the derivative of the curve, the points where the derivative equals zero correspond to the extrema of conductance. By comparing the conductance values at each extremum, the maximum conductance can be determined. The frequency at the maximum conductance point represents the resonant frequency $\omega_{s}=2\pi f$, while the equivalent resistance $R_{s}$ is the reciprocal of the maximum conductance $1/G_{max}$.

There are several calculation methods for the extraction of static capacitance. The expression for the susceptance at frequencies away from the resonance is $B=\omega C_{P}\left(\varepsilon \right)$. So, the value of the static capacitance $C_{P}\left(\varepsilon \right)$ can be obtained by fitting a curve of the susceptance versus frequency at frequencies away from the resonance, or the static capacitance at 1 kHz can be used as the value of $C_{P}\left(\varepsilon \right)$. As shown in Figure 6b, a straight line is fitted using the 20–23 kHz susceptance curve away from the resonance frequency, where the slope of the fitted line is r and the static capacitance is r/2π.

After completing the preprocessing, the system is able to calculate ρ, η, and ε of the transformer oil to be measured according to the resonant frequency, equivalent resistance, and static capacitance of the quartz tuning fork sensor, combined with the Equations (7)–(9) of the quartz tuning fork sensor model. The resonant frequency, equivalent resistance, and static capacitance are obtained by conductance and susceptance spectra extraction.

#### 3.2.5. Data Output

After calculating ρ, η, and ε, the system outputs each of these three parameters via the CAN communication protocol. Through the above-designed program flow, the system realizes the data acquisition and analysis and processing of the quartz tuning fork conductivity spectrogram, and obtains the important parameters such as ρ, η, and ε of the transformer oil to be measured. The whole measurement process is very rapid and can be completed within 3 min, which is of significance for real-time monitoring and provides an efficient and reliable solution for the condition monitoring of transformer oil.

## 4. Testing

### 4.1. Test Platform Construction and Parameter Extraction

**Figure 7:**
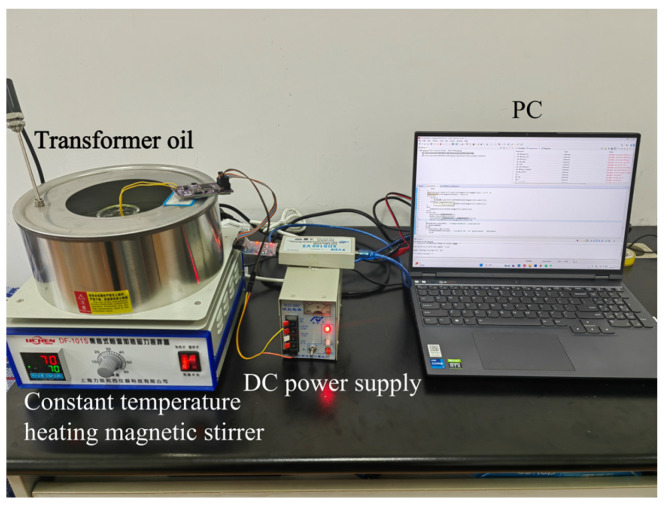
Experimental platform.

Taking the online monitoring of transformer oil as the target, a self-made impedance measurement circuit board, DF-101s collector-type constant temperature heating magnetic stirrer, WD990 DC power supply, and computer were used to build the experimental platform. A photo of the built experimental platform is shown in Figure 7. Transformer oil was then poured into a beaker placed in a constant temperature heating magnetic stirrer filled with water, the temperature of the transformer oil was controlled by the constant temperature heating magnetic stirrer, and the quartz tuning fork was connected to the homemade circuit board and placed into the transformer oil.

Before starting the measurement, the parameters of the quartz tuning fork sensor air equivalent circuit elements needed to be extracted. In this step, the quartz tuning fork with added leads needs to be placed in air. To ensure the accuracy of the measurements, a high-precision impedance analyzer (IM3570) was used to obtain its conductivity spectrum. The impedance analyzer captures changes in the electrical characteristics of the quartz tuning fork under weak vibrations to obtain parameter values for each component in the equivalent circuit. In order to eliminate the influence of temperature fluctuations on the experimental results, the laboratory was maintained at a room temperature within the range of 25 ± 0.1 °C through air conditioning. At the same time, a type of transformer oil was applied to calibrate the normal stress coefficient M, tangential stress coefficient N, and $dC_{P}/d\varepsilon$ coefficients of the quartz tuning fork. After measurement and control, the parameter values of the seven equivalent circuit components were obtained, as shown in Table 2.

### 4.2. Temperature Experiments

The temperature of transformer oil rises during normal operation. This temperature change not only affects the viscosity and density of the transformer oil, but also the resonant frequency of the quartz tuning fork. Calculations of density and viscosity are related to resonant frequency, so the effect of temperature on the resonant frequency of the quartz tuning fork must be considered when analyzing the density and viscosity of transformer oil.

In the temperature range from room temperature to 450 K, the resonant frequency of a quartz tuning fork shows an approximately linear relationship with temperature [28]. This linear relationship provides us with an effective method to correct measurement errors due to temperature variation. By monitoring the temperature of the transformer oil in real time and utilizing the known frequency–temperature characteristic equation (Equation (10)), we can make real-time corrections to the resonant frequency of the quartz tuning fork to obtain more accurate values of transformer oil density and viscosity:

(10) $$f=f_{0}+\alpha \left(T-T_{0}\right)$$

where f and $f_{0}$ are the resonant frequencies at temperatures T and $T_{0}$, respectively, and α is the first order frequency–temperature coefficient.

The quartz tuning fork is placed in the air, and the temperature is controlled by a DF-101s collector-type thermostatically heated magnetic stirrer. The temperature range of the measurements is 25–95 °C, and the quartz tuning fork’s resonance frequency is measured every 10 °C with a sweeping frequency range of 32.65–32.85 kHz and a sweeping interval of 1 Hz.

**Figure 8:**
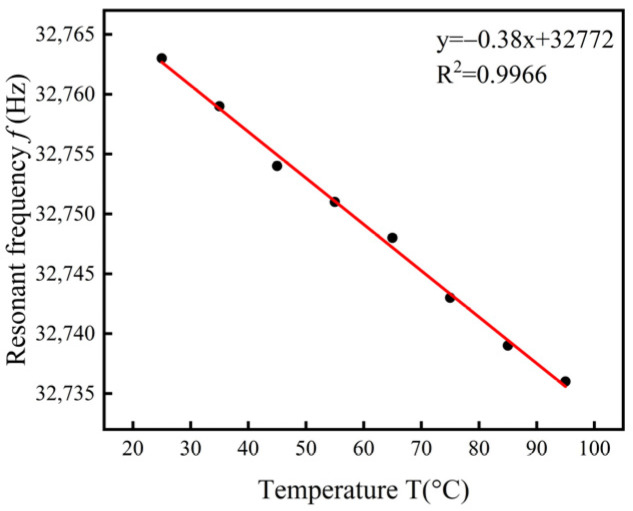
Temperature–frequency curve of a quartz tuning fork.

The resonant frequency of the quartz tuning fork is shown in Figure 8. It can be seen that the frequency–temperature characteristics of the quartz tuning fork used in this paper are basically linear, with the first-order frequency–temperature coefficient α  = −0.38 Hz/°C, and the reference temperature $T_{0}$ = 25 °C. Since the calculation of density and viscosity is related to the resonance frequency, the frequency of the quartz tuning fork is temperature compensated by applying Equation (11) in the calculation of density and viscosity:

(11) $$f^{\prime }=f-\alpha \left(T-T_{0}\right)$$

where $f^{\prime }$ is the resonant frequency after temperature compensation and f is the resonant frequency when the liquid ambient temperature is T.

### 4.3. Repetitive Experiment

**Figure 9:**
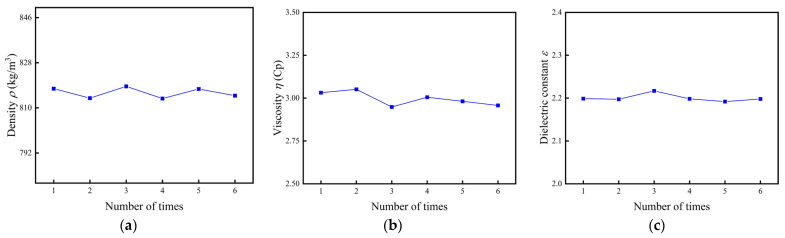
Repeatable experiment of density, viscosity, and dielectric constant. (**a**) Density; (**b**) viscosity; (**c**) dielectric constant.

Repeatable measurements of the conductivity profile of a quartz tuning fork measurement system in transformer oil at 90 °C were made and the results are shown in Figure 9, including resonant frequency, equivalent resistance, and static capacitance values. Relative errors of repeatability for ρ, η, and ε were calculated and evaluated by using the standard deviation Formula (12) and relative error Formula (13). After several repetitions of measurements and calculations, a relative error of 0.34% was obtained for density, 1.76% for viscosity, and 0.51% for dielectric constant, all of which are less than 2%:

(12) $$\Delta =\sqrt{\frac{1}{n-1}\sum_{i=1}^{n}{\left(X_{i}-\bar{X}\right)}^{2}}$$

(13) $$\gamma =\frac{\Delta }{\bar{X}}\;\times \;100\%$$

where Δ denotes the standard deviation, n denotes the number of measurements, $X_{i}$ denotes the i-th measurement, $\bar{X}$ is the mean of measurements, and γ represents the relative error.

### 4.4. Comparison Experiment and Application

**Figure 10:**
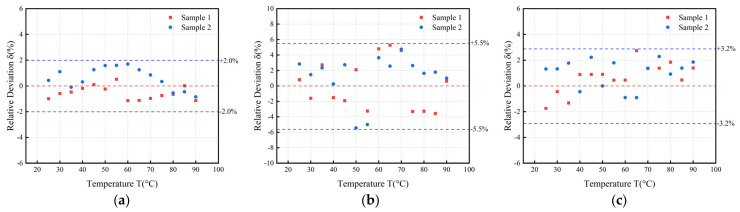
(**a**) Relative error of density measurements; (**b**) relative error of viscosity measurements; (**c**) relative error of dielectric constant measurements.

To validate the error of the designed system, two transformer oil samples were measured using both the standard sensor FPS2800B12C4 and the quartz tuning fork sensor measurement system at intervals of 5 °C from 25 °C to 90 °C. The measurements were conducted with both the standard sensor FPS2800B12C4 and the quartz tuning fork sensor system. By comparing the measurement results obtained from the quartz tuning fork detection system with those from FPS2800B12C4, the relative errors in density, viscosity, and permittivity were derived, as shown in Figure 10.

Before each test of a different oil sample, the quartz tuning fork was cleaned with alcohol and rinsed with deionised water to ensure that residues from the previous test did not affect the subsequent measurement. After cleaning, oil-absorbent paper was used to carefully blot the oil from the surface of the probe, ensuring a clean and dry testing environment. Additionally, sufficient time was allowed between each oil sample test for the tuning fork probe to return to ambient temperature, ensuring that each test was conducted under identical conditions.

The measurements of the quartz tuning fork detector were highly consistent with those of the standard sensor, demonstrating a very high degree of accuracy. In the measurements of density, viscosity, and dielectric constant, the errors were kept at a low level, less than 2.00%, 5.50%, and 3.20%, respectively. 

Relative to the previous results measured using an impedance analyzer [20], the measurement errors of all these three parameters have been reduced, mainly due to the following aspects: (1) The accuracy of the resonance frequency has been improved by using a smaller measurement interval of 1 Hz compared to the previous interval of 6.25 Hz. (2) The extraction of capacitance parameters has been optimized by using the method of fitting a straight line instead of measuring a single point, which improves the effect of static capacitance extraction.

Measurements of viscosity present higher errors compared to measurements of density and dielectric constant. There are several sources of error:

(1) The error ranges of the sensor FPS2800B12C4 are different when measuring different physical quantities. Specifically, the error range for density measurement is ±3%, for viscosity measurement is ±5%, and for dielectric constant measurement is also ±3%. Since viscosity measurement requires higher response speed and stability of the sensor, its error range increases accordingly.
(2) The precision limitation of the impedance measurement chip is also an important possible cause of error. As one of the key components in the experiment, the precision of the impedance measurement chip directly determines the accuracy of the measurement results. By analyzing Equations (7) and (8), the density is mainly related to the resonance frequency, and the viscosity is affected by the resonance frequency and the equivalent resistance at the same time, and there is a certain limitation on the accuracy of the equivalent resistance in the chip measurements, whereas the resonance frequency scanning interval can be very short, so the error of the viscosity will be higher than that of the density.

**Figure 11:**
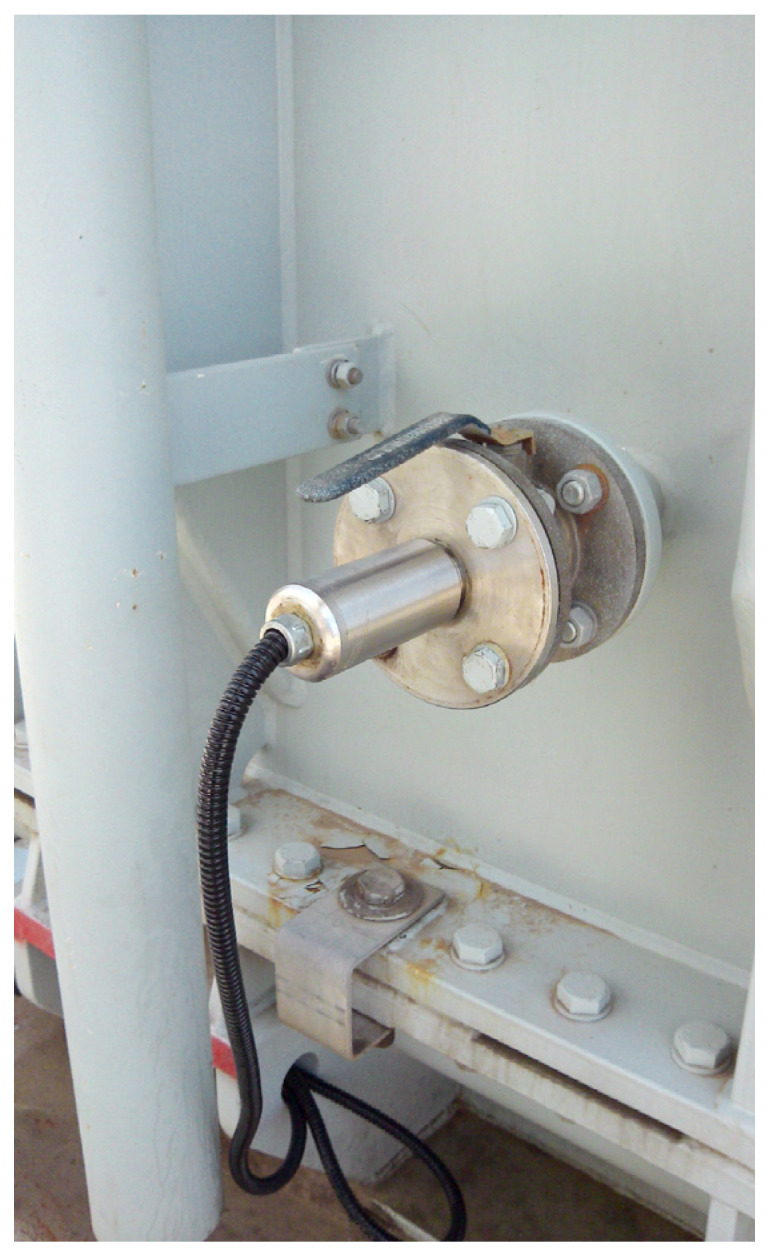
On-site operation diagram.

After completing the error analysis of the measurement system, the online monitoring of transformer oil was carried out as shown in Figure 11. The designed system was installed into the SFPZ-80000/220 oil-immersed power transformer and then the online measurement was carried out and the results compared with the offline operating conditions. The results are shown in Table 3.

As can be noted from Table 3, comparing the offline and online measurements, it can be seen that the data are stable, and the linearity and system reliability are good. The error of the ρ is basically at 1%, the error of η and ε are less than 5% and 2%, respectively. The online and offline operation results show that the system meets the requirements of the condition maintenance system for online monitoring accuracy and real-time detection.

## 5. Discussion

A quartz tuning fork-based system for simultaneous measurement of density, viscosity, and dielectric constant of liquids was designed and implemented by measuring resonant frequency, equivalent resistance, and equivalent capacitance. The resonant frequency was obtained by a frequency sweep circuit, the equivalent resistance was obtained by the reciprocal of the conductance at the resonant frequency, and the equivalent capacitance was obtained by fitting the electrodynamic curve to the non-resonant frequency.

In order to achieve the above measurement objectives, a supporting embedded hardware circuit and software program was designed. This system can efficiently complete the acquisition, processing, and analysis of impedance and conductivity parameters, thus ensuring the accuracy and real-time performance of the simultaneous measurement of density, viscosity, and dielectric constant. 

In order to verify the actual performance of this sensor, an experimental platform was built and tested. The experimental results show that the sensor achieves an error range of 5.50%, 2.00%, and 3.20% in the measurement of viscosity, density, and dielectric constant, respectively. This accuracy not only meets the performance standard of the current sensor FPS2800B12C4, but even exceeds it in some aspects. This proves the usefulness of this sensor in the field of transformer oil parameter measurement.

The small size and light weight of the quartz tuning fork itself makes it easy to miniaturize the entire sensor system, making it easier to install and deploy in limited spaces such as transformers. Measurements of the measuring system installed in the transformer were carried out both offline and online. The results show that the error of the ρ is basically at 1%, and the error of η and ε are less than 5% and 2%, respectively. The results are of good reliability and comparable to the data measured offline.

The main contribution of this article includes the following:

(1) A simultaneous online measurement system has been designed for monitoring the density, viscosity, and dielectric constant of transformer oil during operation, which can also be useful in industrial production due to its suitable size and installation method.
(2) The method of parameter extraction has been optimized to improve the accuracy of the resonance frequency, equivalent resistance, and equivalent capacitance for the measurement system and has been installed in the transformer for online measurement.
(3) It provides a solution for real-time online inspection of transformer oil, as well as a new path for the application of quartz tuning fork crystal resonators in the field of sensors, which is expected to play a role in a wider range of fields, including, but not limited to, industrial automation, environmental monitoring, and medical diagnostics.

## Figures and Tables

**Table 1:** Comparison of impedance measurement methods.

Measurement Methods	Impedance Measurement Range	Applicable Frequency Range	Advantages	Drawbacks
Vector current-voltage method	3 mΩ~100 MΩ	4 Hz~110 MHz	Higher precision, simple to implement	Phase is difficult to measure accurately
Network analysis method	Near 50 Ω	5 Hz~3 GHz	Suitable for high frequency measurements	Limited impedance test range
Resonance method	1 Ω~10 MΩ	10 kHz~70 MHz	Ideal for measuring high Q components	Low accuracy, requires tuning
Automatic balanced bridge method	20 Ω~110 MΩ	20 Hz~100 MHz	Wide impedance, wide frequency range	Complex circuit design

**Table 2:** Extraction results of quartz tuning fork model parameters.

Parameters	$R_{0}\left(k\Omega \right)$	$L_{0}\left(H\right)$	$C_{0}\left(pF\right)$	$C_{P}\left(pF\right)$	M	N	$dC_{P}/d\varepsilon$
value	445.097	6378	0.0037	3.24	2125.63	27,826.51	0.757

**Table 3:** Online and offline measurement results.

Dates	Typology	Density $\rho (kg/m^{3})$	Viscosity η(Cp)	Dielectric Constant ε
20 December 2022	online	821.62	3.18	2.15
	offline	827.06	3.07	2.18
20 February 2023	online	823.55	3.24	2.16
	offline	830.53	3.26	2.14
20 April 2023	online	828.65	3.30	2.18
	offline	835.42	3.27	2.16
19 June 2023	online	833.46	3.47	2.19
	offline	837.42	3.30	2.20
19 October 2023	online	837.54	3.51	2.19
	offline	841.44	3.50	2.21
19 December 2023	online	840.61	3.56	2.20
	offline	847.14	3.52	2.24

## Data Availability

Data are contained within the article.
